# Masticatory biomechanics in the rabbit: a multi-body dynamics analysis

**DOI:** 10.1098/rsif.2014.0564

**Published:** 2014-10-06

**Authors:** Peter J. Watson, Flora Gröning, Neil Curtis, Laura C. Fitton, Anthony Herrel, Steven W. McCormack, Michael J. Fagan

**Affiliations:** 1 Medical and Biological Engineering Research Group, School of Engineering, University of Hull, Hull HU6 7RX, UK; 2 Musculoskeletal Research Programme, School of Medicine and Dentistry, University of Aberdeen, Aberdeen AB25 2ZD, UK; 3 Centre for Anatomical and Human Sciences, Hull York Medical School, University of York, York YO10 5DD, UK; 4 Département d'Ecologie et de Gestion de la Biodiversité, Muséum National d'Histoire Naturelle, Case postale 55, Paris Cedex 5 75231, France; 5 Evolutionary Morphology of Vertebrates, Ghent University, K.L. Ledeganckstraat 35, 9000 Gent, Belgium

**Keywords:** multi-body dynamics analysis, rabbit skull, bite force, mastication

## Abstract

Multi-body dynamics is a powerful engineering tool which is becoming increasingly popular for the simulation and analysis of skull biomechanics. This paper presents the first application of multi-body dynamics to analyse the biomechanics of the rabbit skull. A model has been constructed through the combination of manual dissection and three-dimensional imaging techniques (magnetic resonance imaging and micro-computed tomography). Individual muscles are represented with multiple layers, thus more accurately modelling muscle fibres with complex lines of action. Model validity was sought through comparing experimentally measured maximum incisor bite forces with those predicted by the model. Simulations of molar biting highlighted the ability of the masticatory system to alter recruitment of two muscle groups, in order to generate shearing or crushing movements. Molar shearing is capable of processing a food bolus in all three orthogonal directions, whereas molar crushing and incisor biting are predominately directed vertically. Simulations also show that the masticatory system is adapted to process foods through several cycles with low muscle activations, presumably in order to prevent rapidly fatiguing fast fibres during repeated chewing cycles. Our study demonstrates the usefulness of a validated multi-body dynamics model for investigating feeding biomechanics in the rabbit, and shows the potential for complementing and eventually reducing *in vivo* experiments.

## 1. Introduction

Multi-body dynamics (MDA) is a powerful computational technique, developed for engineering applications, which has recently been applied to a number of biological problems. It is becoming increasingly popular in the field of biomechanics and has already been used to investigate the skulls of extant species, including mammals [1–6] and reptiles [7–9] as well as extinct species [10]. Through simulation of feeding, it is possible to estimate biomechanical parameters such as muscle activations and forces, joint reaction forces, bite forces and jaw movement—parameters that are often difficult or near impossible to measure experimentally.

Once available, the predicted biomechanical loading of a skull can then also be used in conjunction with finite-element analysis (FEA), to further investigate its form and function [3,5,11,12]. However, as with all modelling approaches, it is important to validate that an MDA model behaves in a physiological manner. Previous studies have successfully validated their models through comparing predicted bite forces [2,7,9,13], muscle activations [4] and jaw movements [1] with experimental measurements.

A validated MDA model of the rabbit skull has the potential to develop our understanding of the biomechanics occurring during mastication. Such information can be used in the field of medical research, where it is one of the most commonly used animals [14]: for example, implants are often evaluated through implantation into various regions of the rabbit skull [15–18]; cheek teeth are extracted in order to test bone grafts [19] and to examine the influence of biomaterials on the bone healing process around the tooth sockets [20,21]; the effect of tooth loss on the histochemical composition of the temporomandibular joint (TMJ) cartilage and disc [22], and histology of the condyle [23] have also been reported; and the rabbit TMJ has been surgically altered to investigate the effects of discectomy [24,25] and implantation of disc replacements [26].

A detailed understanding of the healthy rabbit masticatory system is also a prerequisite for diagnosis and treatment of dental disease in rabbits [27]. Although it is one of the most common diseases reported with the rabbit [27,28], the cause remains a matter of debate. Computational modelling can examine theories which link dietary change to a disruption to the eruption of incisor and cheek teeth and subsequent malocclusion. One current theory relates the disease to reduced dental wear owing to fewer chewing cycles [29], whereas another states it is the consequence of metabolic bone disease induced by the lowered intake of calcium and vitamin D [30]; possibly it is a combination of the two [31].

Numerous studies have investigated the form and function of the rabbit feeding apparatus, leading to detailed descriptions of the anatomical structure of individual masticatory muscles [32,33], along with estimation of their sarcomere lengths [34,35]. Muscle activations have been recorded via electromyography (EMG) during consumption of various foods [32,34,36–39], and when the cortical masticatory area and trigeminal motor nucleus are electrically stimulated [40–43]. EMG has also been used to analyse the function of the masticatory muscles in juvenile rabbits during growth [44,45], whereas radiotelemetric devices have measured their daily burst activities [46,47]. Detailed descriptions of jaw movements during mastication of various foods have been reported through the use of cineradiography [32,36,37,48]. Other biomechanical forces generated during mastication, such as joint torques [49], mandibular bone strains [50,51] and bite forces [40,42], have also been recorded experimentally.

Weijs *et al*. [33] reported the first attempt to analyse the biomechanics of the rabbit skull through the calculation of bite forces achieved at various gape angles using vector analyses, within both adult and juvenile rabbits. However, the method employed to calculate resultant muscle forces led to very high bite forces, producing minimal values of approximately 180 N (incisor biting) and approximately 450 N (molar biting) within the adult. Similar calculations have also been performed to analyse changes in the masticatory system during postnatal development [52].

Biomechanical modelling of the rabbit skull must consider movement of the mandible in six degrees of freedom during mastication of different foods. During jaw closing, the working side mandibular condyle moves posteriorly, relative to a stationary balancing side condyle, causing a rotation of the jaw to the working side in order to achieve molar occlusion [32–34,37,48]. Weijs & Dantuma [32] reported two different modes of a subsequent power stroke when observing rabbits consuming hay, pellet and carrot: a crushing movement where the jaw maintains a rotation to the working side (occurs in carrot and frequently in pellet mastication), and a shearing movement where the jaw rotates back to the midline, with an occasional slight over-rotation to the balancing side (occurs in hay and sometimes in pellet mastication). Therefore, as the muscle lines of action vary between the two power strokes, muscle recruitment will be altered during mastication of different foods.

Rabbit mastication is initiated through the collection of a food bolus by the incisors. When consuming tough or large food objects, this is often preceded by gnawing of the incisors to break off small pieces. The bolus is then transported to the molars where it is reduced further through crushing or shearing, or a combination of both (such as the pellet mastication reported by Weijs & Dantuma [32]). Wild rabbits are herbivores with a diet that consists predominately of grasses and forbs [53], although during the winter season they may also feed on bark and needles. Because a combination of incisor and molar biting (crushing and shearing) is used in the mastication of these foods, it is expected that use of these different teeth is optimized to minimize the energy required for mastication.

Despite the wealth of studies that have analysed the rabbit masticatory system, there has been no attempt to model the rabbit skull with MDA. Computational modelling such as MDA enables not only a more accurate prediction of maximum bite forces, but also enables an estimation of the forces associated with the mastication of commonly consumed foods with different material properties. MDA can also provide insights into the biomechanical differences between molar shearing and crushing, and thus the capabilities of the masticatory system to consume a range of different foods can be analysed.

This paper presents the first application of MDA to analyse the biomechanics of the rabbit masticatory system. The aim of this paper is twofold: firstly, to construct an MDA model through the combination of magnetic resonance imaging (MRI) and micro-computed tomography (µCT) scan data, with validation of the model by comparison with experimentally measured bite forces; second, to demonstrate the potential of the model to increase our understanding of masticatory biomechanics through a comparison of the mechanical differences between incisor biting and molar crushing and shearing.

## 2. Material and methods

### 2.1. Bite force measurements

An isometric Kistler force transducer (type 9203, Kistler, Winterthur, Switzerland) mounted on a purpose-built holder and connected to a Kistler charge amplifier (type 5058 A) was used to measure bite force [54]. Five rabbits were caught in the wild at la Réserve Naturelle Nationale de St Quentin en Yvelines, France, during an annual culling programme, and bite forces were measured immediately after capture. When restrained, animals bit defensively, and three sessions of bite force measurements were undertaken for each animal with minimally three bites at the incisors during each session. The measurements were taken with a gape of approximately 5–6 mm. After the bite force measurements were complete, the animals were euthanized.

### 2.2. Visualization of the masticatory system

**Figure 1.**
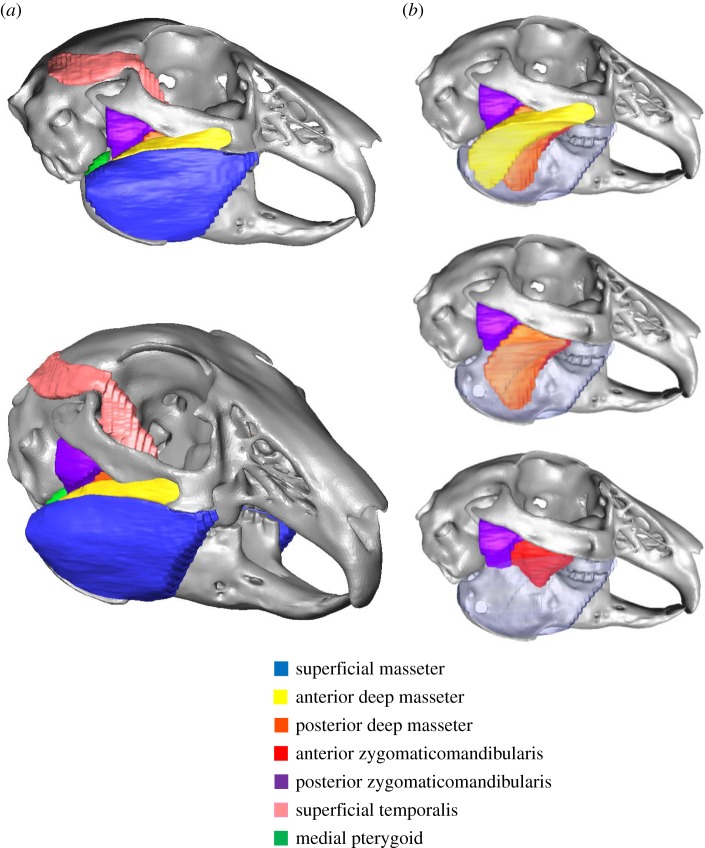
Visualization of the rabbit masticatory system through combining magnetic resonance imaging and micro-computed tomography scan data: (*a*) lateral and oblique views of the masseter, zygomaticomandibularis and temporalis; (*b*) segmentation of the masseter and zygomaticomandibularis into different layers. The pterygoid muscles were also visualized through this method, but are not shown in these views.

A wild rabbit head was scanned using a Discovery MR750 MRI scanner (GE Medical Systems, USA) with a resolution of 127 × 127 × 1000 µm. This individual was not from the same rabbit group that was used for the bite force experiments. The head was subsequently dissected to identify the origin and insertion sites of the jaw closer muscles. This dissection aided segmentation of the MRI scan data within AVIZO image visualization software (AVIZO v.6.3., Visualization Sciences Group, Inc. USA) to produce volumetric models of each jaw closer muscle (figure 1).

Post-dissection, the cranium and mandible were scanned using an X-Tek HMX 160 µCT scanner (X-Tek Systems Ltd, UK) with a resolution of 48 µm in each direction, and volumetric models of each bone were constructed in AVIZO. Superposition of the volumetric models of the jaw closers upon those of the cranium and mandible created a three-dimensional digital representation of the masticatory system (figure 1). Through the combination of medical imaging data with observations during dissection, it was possible to identify several layers of the masseter muscle and the zygomaticomandibularis muscle (figure 1*b*) [32,33,49].

**Figure 2.**
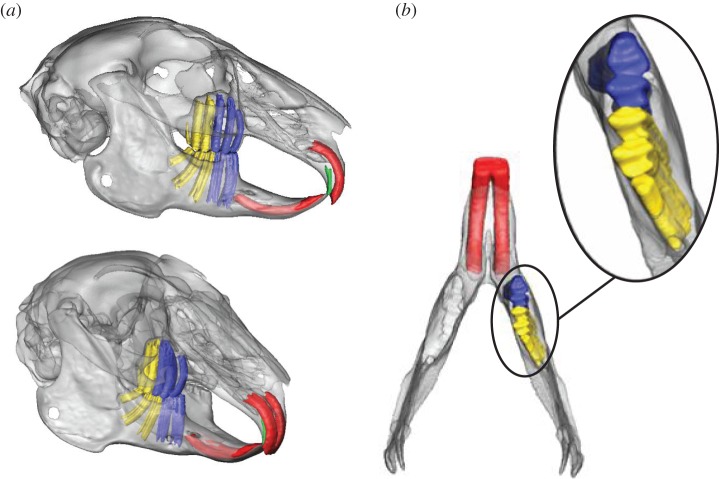
Visualization of the molar and incisor tooth root through micro-computed tomography scan data: (*a*) a lateral and oblique view; (*b*) close up of the tooth roots of the mandible. The premolars (blue) have predominately vertically oriented roots, while the molars (yellow) have a more posterolateral orientation.

The rabbit's teeth were also carefully segmented virtually, allowing the accurate location of bite points during the simulation, and for later incorporation in FEA. The upper jaw of the rabbit contains three premolars and three molars, whereas the lower jaw has two premolars and three molars [32] (figure 2). Through visualization of the tooth roots from the µCT data, it was observed that the premolars of the lower jaw have predominately vertically orientated roots. In comparison, while the molar roots in the maxillae are also vertical, they have a more posterolateral orientation in the mandible (figure 2).

### 2.3. Multi-body dynamics modelling

#### 2.3.1. Model construction

An MDA model was created through importing volumetric models of the cranium and mandible into ADAMS 2013 (MSC Software Corp. USA). The mandible was modelled as a movable part, whereas the cranium was fixed. To allow a realistic range of motion at the TMJ, contact surfaces between the mandible and cranium were defined. The mass and inertial properties of the mandible were calculated within ADAMS based on volume and a standard tissue density of 1.05 g cm^−3^ [2].

**Figure 3.**
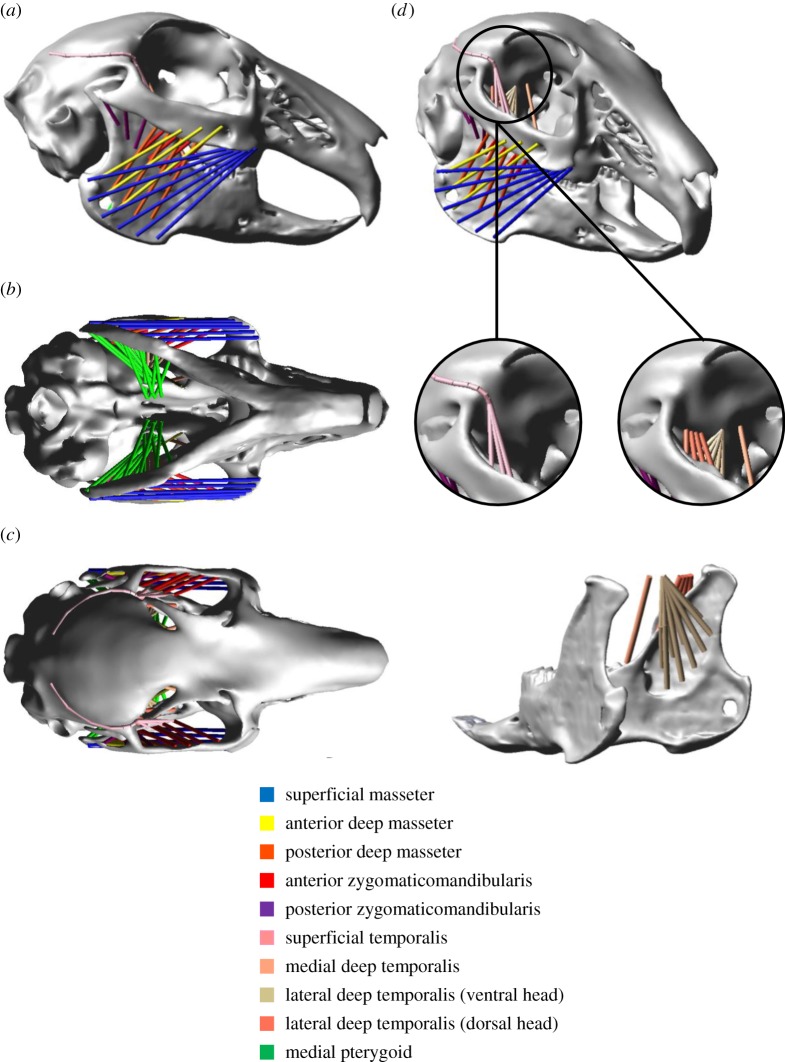
The multi-body dynamics model of the rabbit skull: (*a*) lateral, (*b*) inferior and (*c*) superior views of the masseter, zygomaticomandibularis, temporalis and pterygoid; (*d*) representation of the temporalis in superficial and deep parts. The lateral pterygoid was also included in the model, but is not visible in the depicted views.

Each muscle was modelled as a series of strands in order to capture the differing fibre directions present within a single muscle (figure 3), based on observations from both manual dissection and segmentation of the MRI data (figure 1). The temporalis was modelled in superficial and deep parts (figure 3*d*) using the descriptions of Weijs & Dantuma [32]. The anatomy of the jaw closers was replicated faithfully through inclusion of the individual parts of the masseter (superficial, anterior deep, posterior deep), zygomaticomandibularis (anterior and posterior), temporalis (superficial and deep) and pterygoid (medial and lateral) muscles. The nomenclature of the masticatory muscles in this paper is in accordance with the descriptions of Druzinsky *et al*. [55] (see electronic supplementary material, appendix S1, for explanation of how this nomenclature relates to terminology used by Weijs & Dantuma [32]). During analysis, the forces produced by superficial and anterior deep parts of the masseter were grouped (termed superficial masseter—as also performed by Weijs & Dantuma [32]). The masticatory system was completed by including a jaw opener (digastric muscle), using the origin and insertion sites detailed by Weijs *et al*. [33]. The final model contained a total of 150 strands (75 on each side). Muscle wrapping has been shown to increase the accuracy of MDA model predictions [9], therefore it was also employed here to enable accurate fibre excursions and to prevent muscle–bone intersections. This was particularly important for modelling the superficial temporalis and medial pterygoid (figure 3). For coordinates of muscle origin/insertion and via points, see the electronic supplementary material, appendix S2.

A maximum muscle force was assigned to each jaw opening and closer muscle, calculated by multiplying each muscle's physiological cross-sectional area (PCSA) with a constant muscle stress value. The PCSA of each muscle was calculated via the method described by Anapol & Barry [56]. The mass of each jaw closer was taken directly after dissection, although their fibre characteristics (i.e. length, pennation angle) were not measured. Therefore, the fibre lengths reported by Weijs & Dantuma [32] were used, but, because no published pennation angles were available, pennation angles of zero were assumed. However, in a recent study with a lizard skull, Gröning *et al*. [9] demonstrated that inaccuracies in pennation angle only had a negligible effect. Unfortunately, owing to difficulties separating the posterior deep masseter and the anterior zygomaticomandibularis, their independent masses could not be measured. Therefore, it was estimated using muscle volume (taken from the MRI segmentation) and specific density. As the temporalis was not dissected into different parts, the fibre length of the deeper layers (which are shorter than those of the superficial layers) was used in calculating a PCSA for the whole muscle. Although this causes an overestimation of the maximum force within the superficial temporalis, it was expected to have minimal effect on the bite force due to the small size of the muscle. Table 1 presents the calculated PCSA of each jaw closer, along with their maximum force when using a muscle stress of 25 N cm^−2^ [57]. This muscle stress is an average of the values found within different mammalian muscles.

**Table 1.** Values used to calculate the PCSA and maximum force of the jaw closer muscles.

muscle	mass (g)	fibre length (cm)^a^	PCSA (cm^2^)	max. force (N)
superficial masseter	2.0	0.8	2.4	60.9
posterior deep masseter	0.3^b^	0.7	0.4	10.3
ant. zygo.mandibularis	0.5^b^	0.8	0.6	15.0
post. zygo.mandibularis	0.3	0.7	0.4	10.5
temporalis	1.1	0.8^c^	1.5	37.1
medial pterygoid	1.5	0.6	2.5	62.3
lateral pterygoid	0.3	0.3	0.4	9.8

^a^Taken from Weijs & Dantuma [32].

^b^Estimated using muscle volume and specific density.

^c^Fibre length of the deep temporalis.

The muscle strands were modelled in accordance with a Hill-type muscle model using the parameters of maximum force, activation factor and passive tension. The muscles were activated through the application of the dynamic geometric optimization (DGO) method, which estimates the muscle forces (taking into account the instantaneous strand orientations) to make the jaw follow a specific motion; for a detailed description of the DGO method, see Curtis *et al*. [8]. However, the DGO algorithm was expanded to permit mediolateral movement, thus creating a model whereby the mandible could move freely in six degrees of freedom relative to the cranium. The strands also carried a small passive tension that is naturally developed in resistance to their elongation, which increased exponentially to a maximum of 0.1% of the maximum muscle force. However, this passive tension did not affect muscle activations or bite forces.

#### 2.3.2. Simulation of biting/mastication

**Figure 4.**
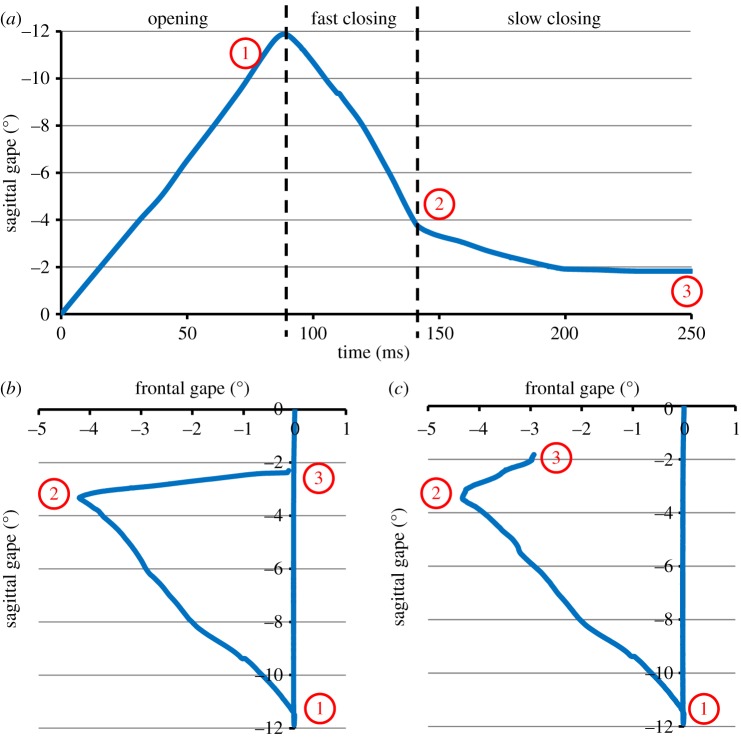
Lateral and frontal gape angles of the jaw against time during molar biting (shearing and crushing): (*a*) gape in the lateral view; (*b*) gape in the frontal view during molar shearing; (*c*) gape in the frontal view during molar crushing (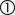 = point of maximal opening of the jaw, marking the end of the opening phase, 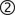 = point at which the jaw contacts the food, marking the end of the fast closing phase, 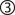 = end of the slow closing phase whereby the food bolus has been compressed to half of its original height). Developed from Weijs & Dantuma [32]. (Online version in colour.)

Three different modes of biting or mastication were simulated: incisor biting, molar crushing and molar shearing. In simulating the molar modes, the DGO algorithm was applied to follow a motion path based on *in vivo* kinematic data from Weijs & Dantuma [32]: namely a maximal 12° gape in the sagittal plane during jaw opening, and a 4° rotation to the working side in the frontal plane during jaw closing (figure 4). During molar shearing, the jaw rotated back to the midline when in contact with the food bolus (figure 4*b*), whereas it maintained a lateral rotation during molar crushing (figure 4*c*). Incisor biting was modelled with the same maximal gape, but with symmetrical rotation about the midline in the frontal plane. Each mode of mastication was modelled with three distinct phases, consistent with the description of Schwartz *et al*. [36] of a reduction bite cycle, i.e. (i) opening phase, (ii) fast closing phase (closing of the jaw until it contacts a food bolus), and (iii) slow closing phase (were the food bolus is crushed/sheared; figure 4). A reduction bite cycle was chosen as it is representative of the midpoint of a chewing cycle (i.e. occurring after the food bolus is transported to the molars, but before it is prepared for swallowing). The duration of each phase was also consistent with literature [36].

Molar biting (crushing and shearing) was simulated as a unilateral bite with the food bolus positioned between the most posterior molars on the right side of the jaw. The food bolus was modelled as two rigid plates separated by a spring element which connected the two parts at a coincident location. A contact with a high friction coefficient was defined between the lower plate and the jaw to ensure there was minimal displacement between the two. The spring element was defined with three orthogonally directed forces, all of which were proportional to the distance between the two plates (i.e. the resistance increased as the bolus was crushed/sheared).

During molar shearing, the spring element was defined, so that the jaw had to overcome a resistance of 20 N in the mediolateral and anteroposterior directions, in order to return to the midline. This aimed to mimic the shear strength of the foods identified by Weijs & Dantuma [32] that required molar shearing, and was within the range observed for wheat and barley straw [58,59]. A maximal resistance of 60 N was defined in the vertical direction in order for the food bolus to be compressed completely.

Simulation of molar crushing aimed to mimic the processing of pieces of carrot [32], with the spring element defined, so the jaws had to overcome a maximal resistance of 100 N in order to compress the food bolus completely. As simulations of molar crushing aimed to replicate the midpoint of the chewing cycle, the food bolus was compressed to roughly half of its original height. This follows the descriptions of Schwartz *et al*. [36] that the upper and lower teeth rarely contact during a reduction bite cycle (i.e. the food is not fully compressed). Consequently, the actual maximal resistance experienced during the simulation was only 50 N. This value is within the range of compressive forces which are required to achieve a break point (determined as the point where there was a reduction in stress) in carrots of similar size (the bolus was 1.3 mm here) [60,61].

When simulating incisor biting, the height of the food bolus was increased (to 2.4 mm), so that the slow closing phase duration of 140 ms could be maintained. The spring element was defined with the same vertical resistance as molar crushing, and once again the food bolus was compressed to half of its original height during the simulation (therefore, a maximum vertical resistance of 50 N was experienced). This enabled a comparison of the mechanical advantage of molar crushing versus incisor biting.

For comparison with recorded *in vivo* bite forces, the food bolus was defined with a significantly high spring element stiffness (to prevent compression in any direction). A simulation was performed with a 5.5 mm gape when the jaw was in contact with the food bolus (to mimic the experimental set-up). The jaw closers were subsequently able to reach their maximum forces (i.e. 100% activation), thus producing the maximum bite force achievable.

## 3. Results

### 3.1. *In vivo* and modelling comparisons

**Figure 5.**
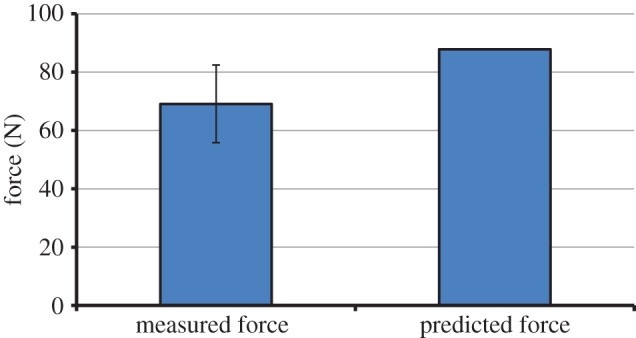
Comparison between measured and predicted maximum incisor bite forces. The error bar indicates ±1 standard deviation of the measurement mean. (Online version in colour.)

Skull size (in terms of length, width and depth) was found to be similar between the modelled individual and the wild group that underwent the bite force experiments (see electronic supplementary material, appendix S3). Measurements of incisor biting yielded an absolute maximum value of 95.2 N across all animals, but an average maximal force of 69.1 N with a standard deviation (s.d.) of ±13.3 N. In comparison, the MDA model predicted a maximum bite force of 87.8 N, which fell above the range of ±1 s.d. of the experimental mean (figure 5), but was lower than the absolute maximum measured force.

### 3.2. Biomechanics of molar and incisor biting

**Figure 6.**
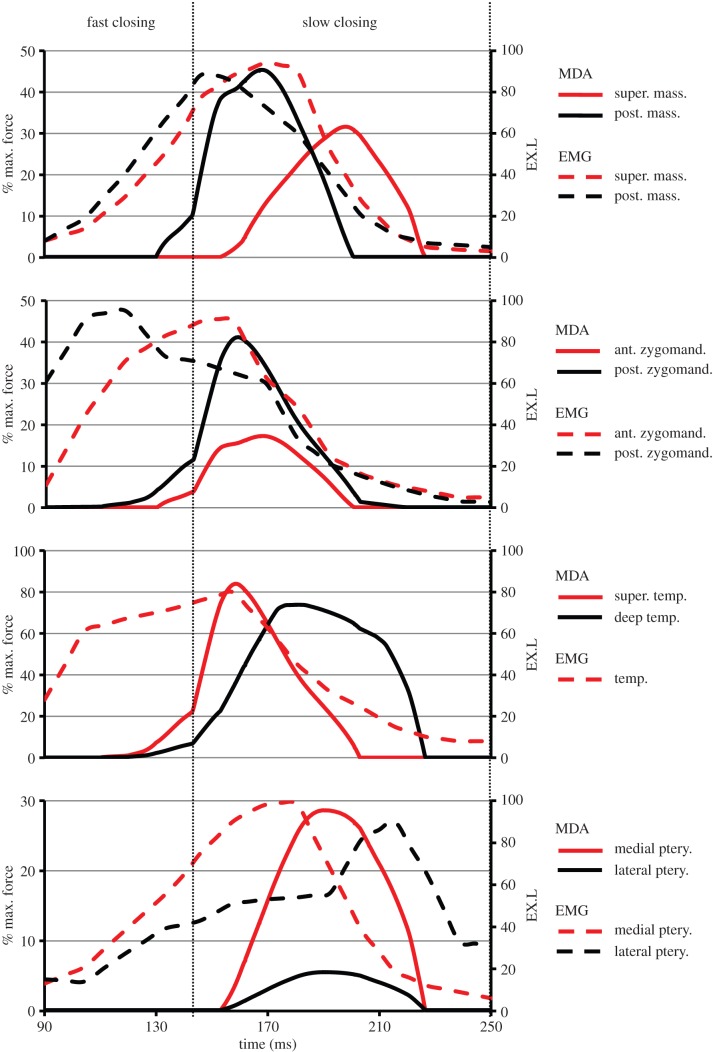
Activation of the working side jaw closer muscles (expressed as a percentage of their maximum force) predicted by the MDA simulation during molar shearing. The muscles responsible for molar occlusion (group 1, see text) activate during the fast closing stage and peak early in the slow closing phase. The muscles responsible for shearing of the food bolus (group 2, see text) activate at the beginning of the slow closing phase and peak shortly afterwards. The EMG profiles reported by Weijs & Dantuma [32] for pellet mastication are also shown (expressed as an excitation level (EX.L)) (note that EMG profile for the temporalis is an average for the whole muscle). (Online version in colour.)

**Figure 7.**
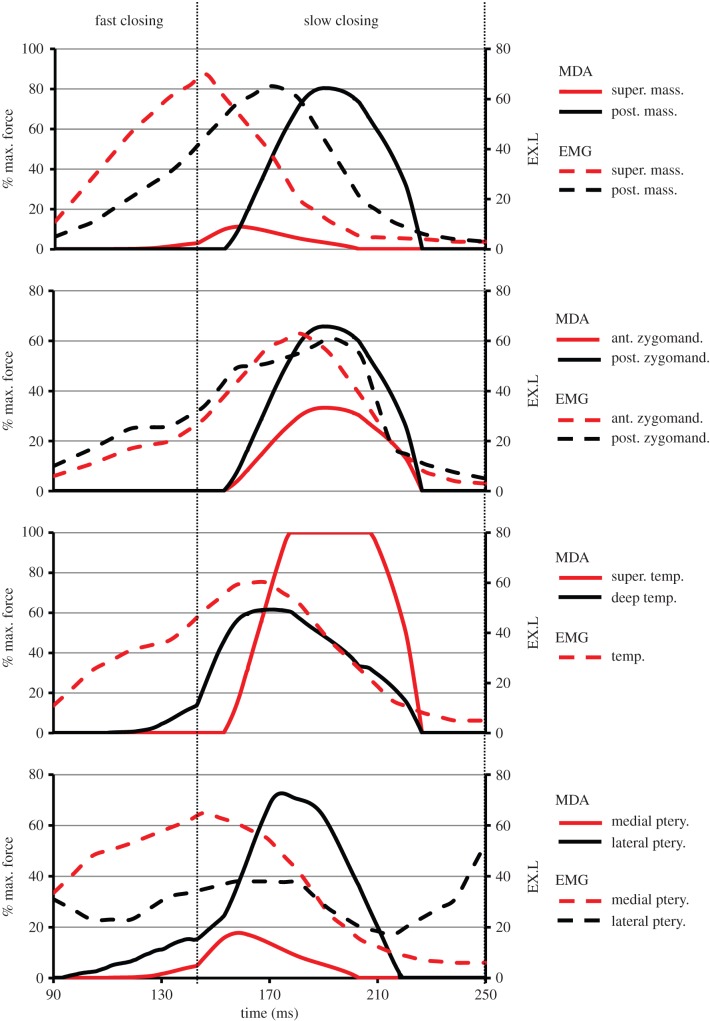
Activation of the balancing side jaw closer muscles (expressed as a percentage of their maximum force) predicted by the MDA simulation during molar shearing. The muscles responsible for molar occlusion (group 1, see text) activate during the fast closing stage and peak early in the slow closing phase. The muscles responsible for shearing of the food bolus (group 2, see text) activate at the beginning of the slow closing phase and peak shortly afterwards. The EMG profiles reported by Weijs & Dantuma [32] for pellet mastication are also shown (expressed as an excitation level (EX.L)) (note that EMG profile for the temporalis is an average for the whole muscle). (Online version in colour.)

The variation in the activation of the jaw closer muscles during the fast and slow closing phases of molar shearing are presented in figure 6 (working side) and figure 7 (balancing side). The DGO algorithm was defined to activate the jaw closer muscles in two specific groups, following descriptions from EMG recordings [32,37]. During the fast closing phase, a group of muscles (group 1) consisting of the working side posterior deep masseter, anterior zygomaticomandibularis, posterior zygomaticomandibularis, superficial temporalis, deep temporalis and the balancing side superficial masseter, medial pterygoid and lateral pterygoid, were activated. These muscles reached peak activation early in the slow closing phase. Owing to their resultant orientation, muscle group 1 causes the working side mandibular condyle to retract, a medial rotation of the jaw and subsequent molar occlusion by the end of the fast closing phase. At the onset of the slow closing phase, a second group of muscles (group 2) consisting of the working side superficial masseter, medial pterygoid and lateral pterygoid, and balancing side posterior deep masseter, anterior zygomaticomandibularis, posterior zygomaticomandibularis, superficial temporalis and deep temporalis, were activated. Muscle group 2 causes the mandibular condyle to protract, and produce rotation of the jaw back to the midline. These muscles reached peak activation half way through the slow closing phase.

Comparison with EMG recordings demonstrates that the jaw closers were activated in a physiological sequence (i.e. in the correct muscle groupings; figures 6 and 7). However, it is important to note that the magnitude of the activation levels are not comparable as the two datasets are normalized to differing measures (MDA profiles are expressed as a percentage of maximum force, whereas EMG data are expressed as an excitation level (EX.L.); see Weijs & Dantuma [32] for definition). It was observed that the majority of EMG profiles achieved peak activations before those of the MDA simulation. Consequently, they attain a higher level of activation by the end of the fast closing phase. In addition, differences between EMG and MDA activation profiles vary among the muscle groups. The largest differences in activation profiles are found in the working side posterior zygomaticomandibularis and lateral pterygoid, along with the balancing side lateral pterygoid. The *in vivo* measurements observed the initial activation of the working side posterior zygomaticomandibularis occurring late in the opening phase, thus it had already developed a high activation level at the beginning of the fast closing stage (figure 6). However, the MDA simulation performed jaw opening through the sole action of the digastrics; therefore, the posterior zygomaticomandibularis did not activate until the fast closing phase (in order to produce molar occlusion). The balancing side lateral pterygoid was observed to assist in jaw opening, thus its EMG profile had a plateau of low activation during the fast and slow closing phases, whereas the MDA simulation recruited this muscle to assist in producing molar occlusion. Possible explanations for these differences could lie in the fact that the two datasets represent slightly different power strokes, and in the methodology of the DGO algorithm (for further explanation, see the Discussion).

**Figure 8.**
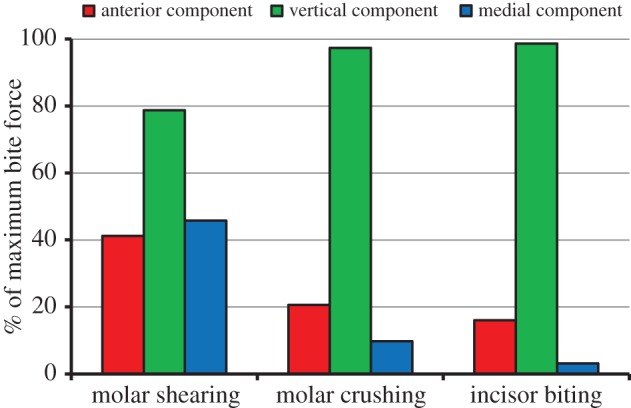
Comparison of the bite force components (expressed as a percentage of the resultant force) predicted during molar shearing and crushing, and incisor biting. (Online version in colour.)

This coordinated activation of muscle groups 1 and 2 generated a bite force that is initially directed vertically. However, as the muscles in the group 1 reached their peak activations and the muscles in the group 2 increased in activation, the shearing components of the bite force became more prominent. Consequently, a resultant bite force of 35.4 N was predicted at the point where the group 2 muscles reached their peak activations. This force had a large vertical component (78.8% of resultant), although there were also marked anterior and medial components (41.2% and 45.8% of resultant, respectively; figure 8).

**Figure 9.**
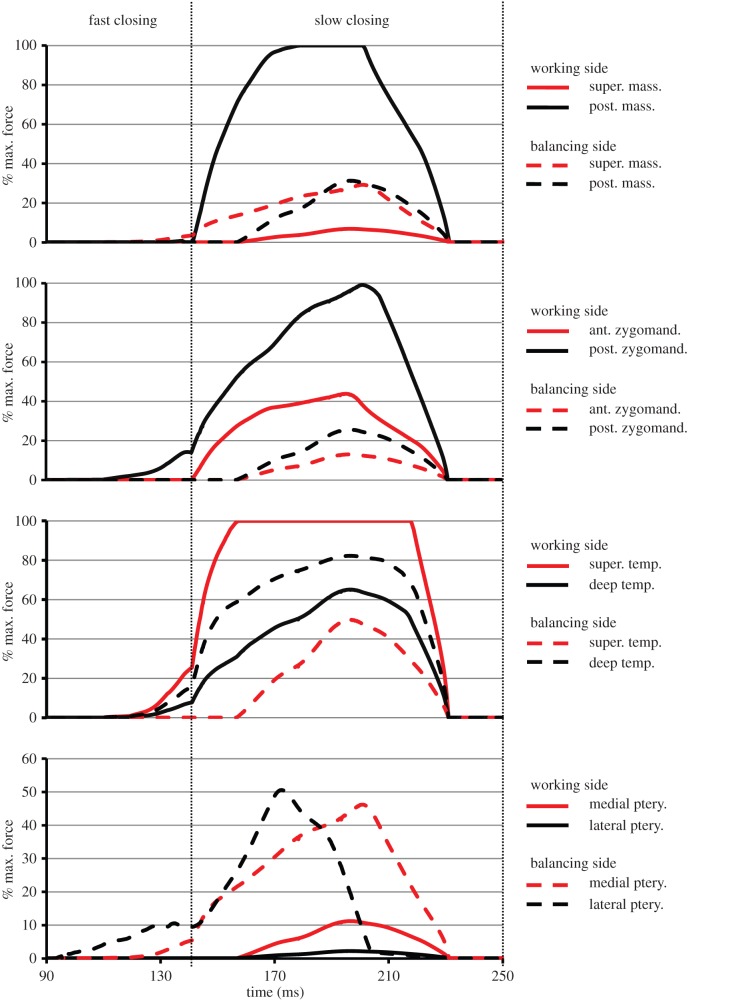
Activation of the jaw closer muscles (expressed as a percentage of their maximum force) during molar crushing. The muscles responsible for molar occlusion (group 1, see text) activate during the fast closing stage and maintain a high force during the slow closing phase. The muscles responsible for stabilizing the retracted working side mandibular condyle (group 2, see text) activate at the beginning and then peak at the midpoint of the slow closing phase. (Online version in colour.)

When simulating molar crushing, the DGO algorithm was once again defined to activate muscle group 1 during the fast closing phase, in order to achieve molar occlusion (figure 9). As these muscles contain fibres that have a strong vertical component, they are also responsible for crushing movements. Therefore, muscle group 1 maintained activation deeper into the slow closing phase, reaching peak levels once the food bolus had been compressed sufficiently. Peak activations generally exceeded those observed during molar shearing, with the working side posterior deep masseter and posterior zygomaticomandibularis displaying the largest increases (54.7% and 58.0%, respectively; figure 9 and table 2). By contrast, muscle group 2 was recruited to stabilize the retracted position of the working side mandibular condyle. Therefore, the peak activations in muscle group 2 during molar crushing were generally lower than those observed with molar shearing, most notably in the balancing side posterior deep masseter and superficial temporalis which reduced by 48.8% and 50.2%, respectively (figure 9 and table 2).

**Table 2.** The peak activation values of the jaw closer muscles (expressed as a percentage of their maximum force) during molar and incisor biting. As incisor biting consists of symmetric jaw opening/closing about the midplane, the majority of muscle activations are the same on both the balancing and working sides.

muscle	molar shearing		molar crushing		incisor biting
	balancing	working	balancing	working	
superficial masseter	11.3	31.6	29.3	6.9	38.7
posterior deep masseter	80.2	45.3	31.4	100.0	100.0
ant. zygo.mandibularis	33.3	17.3	13.1	43.9	70.0^a^
post. zygo.mandibularis	65.7	41.1	25.7	99.1	100.0
sup. temporalis	100.0	83.9	49.8	100.0	100.0
deep temporalis	61.6	73.8	82.3	65.1	100.0
medial pterygoid	17.7	28.6	46.2	11.2	62.3^a^
lateral pterygoid	72.6	5.5	50.6	2.2	11.4^a^

^a^Average activation between the balancing and working sides.

Owing to higher activation of muscle group 1, molar crushing was capable of producing a larger resultant bite force of 51.5 N. This bite force was almost completely attributed to a vertical component (97.4% of resultant), with only minor contributions from the anterior and medial components (20.6% and 9.8% of resultant, respectively; figure 8).

Incisor biting was simulated through defining the DGO to activate all the jaw closer muscles simultaneously, in order to achieve symmetric closing about the midplane. Despite inevitable natural asymmetries in the skull, jaw joints and muscle positions and lines of action, the majority of the muscles produced very similar activations on both sides of the mandible. However, relatively small discrepancies were found between the contralateral anterior zygomaticomandibularis (10.4%), medial pterygoid (3.5%) and lateral pterygoid (13.0%). For these muscles, the average activation between the balancing and working sides has been reported in table 2. Peak activations were reached when the food bolus had been compressed sufficiently, which occurred at the midpoint of the slow closing phase. A resultant bite force of 50.8 N was generated, which was once again generated by a predominant vertical force (figure 8). The only notable difference compared with molar crushing was the lower contribution (albeit slight) of the anterior and medial components. However, in order to generate a similar bite force, the majority of the jaw closer muscles achieved much higher peak activations compared with molar crushing (table 2). The balancing side posterior deep masseter and posterior zygomaticomandibularis displayed the largest increases of 68.6% and 74.3%, respectively. Only the balancing side lateral pterygoid produced a lower peak activation, although this is attributed to the minimal shearing involved during incisor biting.

## 4. Discussion

The rabbit feeding system has previously been investigated via experimental methods such as EMG [32,34,36–39] and cineradiography [32,36,37,48], providing an insight into the complex functioning of the masticatory system during the consumption of different foods. Coordinated recruitment of specific muscle groups is required in order to generate the necessary jaw movement for the shearing and crushing of foods [32,37]. This paper is the first to apply MDA to the rabbit skull and investigate the masticatory system and its biomechanics in detail. This model has successfully combined MRI and µCT scan data of the same individual in order to represent the complex muscle arrangements, and can simulate different modes of mastication.

A particular feature of MDA which increases its potential for modelling skull biomechanics is the ability to model muscles with multiple strands with differing lines of actions (figure 3), allowing an accurate representation of complex muscle anatomy. For example, in this model, the superficial masseter is represented with anterior fibres that are predominately directed vertically, whereas the posterior fibres have a larger horizontal component. Coupled with the application of the DGO algorithm, this enables simulations whereby anterior fibres are used during crushing movement, whereas posterior fibres have increased activity during shearing.

The model was validated through comparison between predicted and experimentally measured incisor bite forces. The MDA model predicted a maximum bite force which was only 18.7 N larger than the average measured force, and only 7.4 N lower than the absolute maximum measured force (figure 5). Previous mathematical modelling has significantly overestimated the maximal bite forces of adult rabbits, reporting values of over 600 N for molar crushing [33], and comparative maximal bite forces of over 400 and 200 N for premolar crushing and incisor biting, respectively.

The maximum bite force predicted in the current study is similar to those measured experimentally, despite the fact muscle fibre pennation angle was ignored. However, Gröning *et al*. [9] recently demonstrated that altering pennation angles had only a minimal effect on bite force predictions, although its inclusion here would lower the maximum force of the jaw closer muscles. Furthermore, the use here of fibre length values from the literature also adds errors into the estimation of maximum muscle forces. It is also noted that the model uses a muscle stress which is towards the lower end of the range of values reported within the literature. Comparison between the predicted bite force and the experimental dataset must also consider the possibility the rabbits did not bite maximally during the force measurements.

When measured EMG profiles and predicted muscle activations are compared, it is clear that peak EMG activity usually occurs earlier in the bite cycle (figures 6 and 7). This can be explained by the implementation of the DGO algorithm, in which muscle activation and hence motion occurs instantly. However, an *in vivo* delay of 13–30 ms is reported in rabbits between the onset of electrical activity and muscle activation [62–64]. Therefore, the predicted muscle force profiles will appear out of phase in comparison with the experimental recordings. In addition, a more detailed comparison of the two is not possible here as they reflect slightly different jaw movements. The simulation of molar shearing consisted of a jaw returning to the midline (figure 4*b*), whereas the EMG measurements were taken during mastication of a pellet, which involves jaw movement that is intermediate between shearing and crushing [32]. Therefore, the difference in muscle recruitment required to produce the two jaw movements, could also account for time shifts between the peak activations.

The potential of the model to analyse the rabbit skull has been demonstrated through investigation of the biomechanical differences between incisor biting and molar crushing and shearing. Consideration of the components of the maximum bite force during molar shearing has highlighted the ability of the masticatory system to process foods through shearing in more than one direction. Although the largest contribution to the resultant bite force is vertical, there are also significant contributions from components in the anterior and medial directions (figure 8). This ensures that the bolus is sufficiently deformed in order to process the food. In comparison, much higher maximal bite forces were produced during molar crushing and incisor biting, which were both achieved through a predominant vertical component (figure 8). Therefore, through alteration in the recruitment of the masticatory system (cf. figures 6, 7 and 9) the biting force is configured to primarily compress the food bolus. As the simulations of molar crushing and incisor biting had to overcome the same resistance in order to compress the food, their maximal bite forces are similar. Table 2 displays the increased muscle activity used during incisor biting in order to overcome the resistance and compress the food bolus. Thus, incisor biting required 64.0% of the total muscle force available throughout the masticatory system, whereas molar crushing used only 35.6%. Therefore, in this model, molar crushing is more efficient at converting muscle force into bite force, when compared with incisor biting (i.e. it is more mechanically efficient). In comparison, molar shearing uses only 27.0% of the total muscle force available throughout the masticatory system. However, it should be noted that this observation is based on an incisor bite characterized by compression of the food bolus, which was simulated to provide a comparable measure with molar and premolar crushing. It does not consider cutting of the food by the incisors, which is their primary function, as it is beyond the scope of this current analysis.

Simulations of premolar crushing were also performed using the same modelling method. In order to generate a maximal bite force of 50.9 N (which was predominately attributed to a vertical component—see electronic supplementary material, appendix S4), the activations of muscle group 1 were slightly larger than those of molar crushing, although the activations of muscle group 2 were similar. Consequently, premolar crushing used 40.5% of the total muscle force available throughout the masticatory system.

The superficial temporalis is the only muscle that reached 100% activation during all three modes of mastication (figures 6, 7, 9 and table 2), although this is likely to be a result of its fairly low maximum force (6.2 N). Additionally, as this muscle has vertically oriented strands attaching to the descending ramus (figure 3*d*), it is recruited in all jaw closing movements, thus reaching maximum activation fairly quickly. The vertical orientation of the strands in the deep temporalis (figure 3*d*), also results in higher activation levels within this muscle compared with others. In comparison, the superficial masseter displayed the lowest activation levels, typically never reaching above 40% of maximum force. This is because it has the highest maximum force of all the masticatory muscles (60.9 N), and the muscle strands vary significantly in their orientation due to its pennate structure (figure 3). Consequently, the superficial masseter is not optimized to any one particular function (such as the temporalis), as it is used anteriorly during jaw closing and posteriorly during shearing movements. However, despite the low activation levels, the superficial masseter still produced some of the largest forces in the masticatory system (owing to its large maximum force).

Simulation of shearing/compression of the food bolus though recruitment of a fairly low percentage of the total muscle force throughout the masticatory system, could indicate potential energy conservation required for rabbit mastication. Schwartz *et al*. [36] recorded the occurrence of 17 consecutive reduction cycles before the pre-swallowing cycles started. Recruiting consistently higher activations of the jaw closers could hamper ability of the masticatory system to perform a series of such cycles. This suggests that the use of the molars is optimized to be mechanically efficient, i.e. more efficient to process a food bolus through a series of cycles with low muscle activations, rather than through fewer cycles with higher muscle activations. Because incisor biting is less effective at converting muscle force into bite force, its use could be optimized more for cutting food into smaller pieces, and not for multicycle biting. These pieces are then transferred posteriorly for further reduction through a series of molar chewing cycles. This is consistent with the theory that during intraoral food processing the rabbit uses its incisors and molars in a coordinated manner to provide the greatest mechanical efficiency. However, note that the link between muscle activation and energy minimization discussed here is not related directly to metabolic cost, which is not considered explicitly in this model.

The MDA simulations modelled a food bolus with a relatively low peak resistance in compression and shear. The resistance was particularly low during molar shearing as it aimed to mimic mastication of foods such as hay or grass. This was reflected in the relative ease at which the mandible was able to shear the food bolus and return to the midline, as highlighted by the low activation levels of some muscles within group 2 (the superficial masseter, anterior zygomaticomandibularis and medial pterygoid all had activation levels below 35% of maximum force; table 2). In contrast, molar crushing experienced a maximum resistance of 50 N during jaw closing (only 2.5 times higher than in shearing), attributing to only the posterior deep masseter, posterior zygomaticomandibularis and temporalis reaching activation levels above 70%. Therefore, it can be concluded that the masticatory system has sufficient strength to crush and shear food of greater resistance than simulated here. Compressive forces ranging between 175 and 305 N are required to generate 50% strain in larger pieces of carrot (10 mm) than modelled here [65]. Therefore, a 4 mm piece that fits between the premolars (measured with a 12° gape) might be expected to require a force of 70–122 N, which is within the range of the values predicted here.

As with all modelling simulations, this model and analysis does have limitations, particularly in the definition of the maximum muscle forces. Calculation of each muscle's PCSA value would ideally consist of data measured from the individual dissected, however, fibre lengths were taken from literature in this study. Similarly, confidence in the bite force measurements and EMG recordings would be improved if they were obtained from the dissected individual. It was also assumed that incisor biting uses the same reduction bite cycle as molar mastication, with the slow closing phase during incisor biting being modelled with the same duration as molar biting. Because the biting simulated compression of the food bolus to half of its original height, the timings of the closing phases will only influence the duration of the muscle force curves, not the maximum activation levels reported in table 2.

In conclusion, this paper has successfully combined MRI and µCT data, together with manual dissection, to construct an MDA of the rabbit skull. Analyses with the model demonstrated that molar shearing is able to deform a food bolus in all three orthogonal directions, whereas molar crushing and incisor biting use a differing muscle recruitment strategy in order to achieve forces which are predominately directed vertically. Simulations also suggest that the masticatory system is optimized to use minimal energy (particularly during molar shearing), performing a series of bite cycles which recruit only a proportion of the total muscle force available. This model also provides biomechanical data for future FEA to reveal strains generated within the rabbit skull during mastication. This computational modelling has potential medical applications, to understand and treat dental disease in the rabbit, and in the reduction of animal testing by offering the potential to investigate surgical interventions and new implant designs *in silico*.

## Supplementary Material

Appendix 1 - Nomenclature of the rabbit masticatory apparatus

## Supplementary Material

Appendix 2 - Muscle origin, attachment and via point coordinates

## Supplementary Material

Appendix 3 - Comparison of skull size between the individual that was modelled and the wild group that underwent the bite force experiments

## Supplementary Material

Appendix 4 - Resultant bite forces and maximum muscle activations when simulating premolar crushing

## Supplementary Material

Appendix 5 - Muscle activation profiles
