# Supplementary material for: Masticatory biomechanics in the rabbit: a multi-body dynamics analysis
Source: J R Soc Interface. 2014 Oct 6;11(99):20140564. doi: 10.1098/rsif.2014.0564 (PMC4233732; doi:10.1098/rsif.2014.0564)
Supplement: Appendix 1 - Nomenclature of the rabbit masticatory apparatus [file rsif20140564supp1.doc]

**Appendix 1**

**Nomenclature of the rabbit masticatory apparatus**

The nomenclature of the masticatory muscles used in this paper was based on the classifications of . The table below illustrates how the employed terminology relates to the nomenclature used by .

| Muscle names in this paper | Muscle names used by |
| --- | --- |
|  |  |
| Superficial masseter | Superficial masseter Part 1a |
| Anterior deep masseter | Superficial masseter Part 1b |
| Posterior deep masseter | Superficial masseter Part 2 |
|  |  |
| Anterior zygomaticomandibularis | Anterior deep masseter |
| Posterior zygomaticomandibularis | Posterior deep masseter |
|  |  |
| Superficial temporalis | Superficial temporalis |
| Deep temporalis | Deep temporalis |
|  |  |
| Medial pterygoid | Medial pterygoid |
| Lateral pterygoid | Lateral pterygoid |

**References**

[1] Druzinsky RE, Doherty AH, De Vree FL. 2011 Mammalian masticatory muscles: homology, nomenclature, and diversification. *Integr. Comp. Biol.* **51**, 224-234. (doi:10.1093/icb/icr067).

[2] Weijs WA, Dantuma R. 1980 Functional anatomy of the masticatory apparatus in the rabbit (Oryctolagus cuniculus L.). *Neth. J. Zool.* **31**, 99-147. (doi:10.1163/002829680X00212).
