# Supplementary material for: Masticatory biomechanics in the rabbit: a multi-body dynamics analysis
Source: J R Soc Interface. 2014 Oct 6;11(99):20140564. doi: 10.1098/rsif.2014.0564 (PMC4233732; doi:10.1098/rsif.2014.0564)
Supplement: Appendix 3 - Comparison of skull size between the individual that was modelled and the wild group that underwent the bite force experiments [file rsif20140564supp3.pdf]

Appendix 3

Comparison of skull size between the individual that was modelled and the wild group that underwent the bite force experiments

The skull size (measured in terms of length, width and depth) of the individual that was modelled was close to that of the wild group used for the bite force experiments. The skull length and width were only slightly lower than the average of the wild group (2.0mm and 1.48mm, respectively), while the depth was slightly higher than the mean (2.62mm).

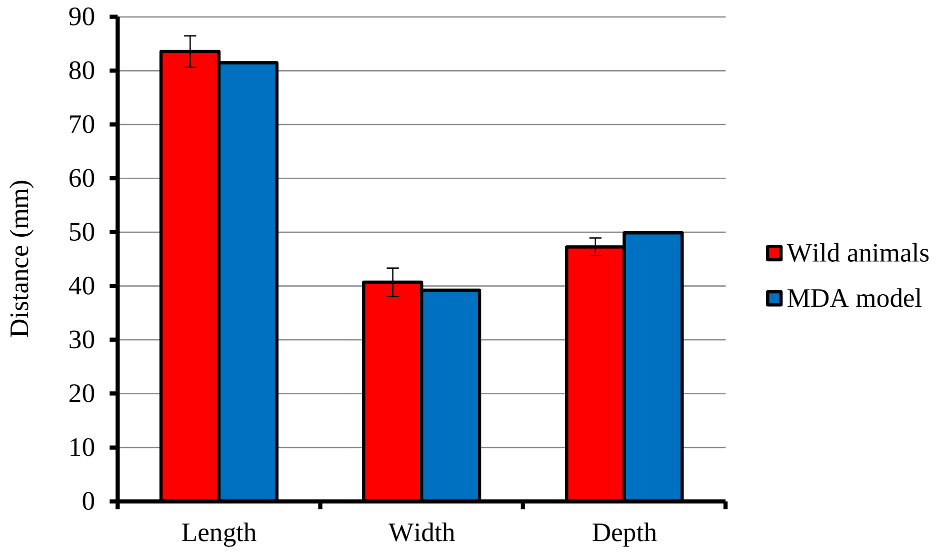

The error bar indicates ± one standard deviation of the measurement mean.
