# Supplementary material for: Masticatory biomechanics in the rabbit: a multi-body dynamics analysis
Source: J R Soc Interface. 2014 Oct 6;11(99):20140564. doi: 10.1098/rsif.2014.0564 (PMC4233732; doi:10.1098/rsif.2014.0564)
Supplement: Appendix 4 - Resultant bite forces and maximum muscle activations when simulating premolar crushing [file rsif20140564supp4.pdf]

Appendix 4

Resultant bite forces and maximum muscle activations when simulating premolar crushing

As with molar crushing, the force required to compress the food bolus to half of its original height was defined as 50 N for premolar crushing. The resultant was predominately directed in the vertical direction (98.1% of resultant), with only small contributions from the anterior (14.1% of resultant) and medial (12.9% of resultant) components.

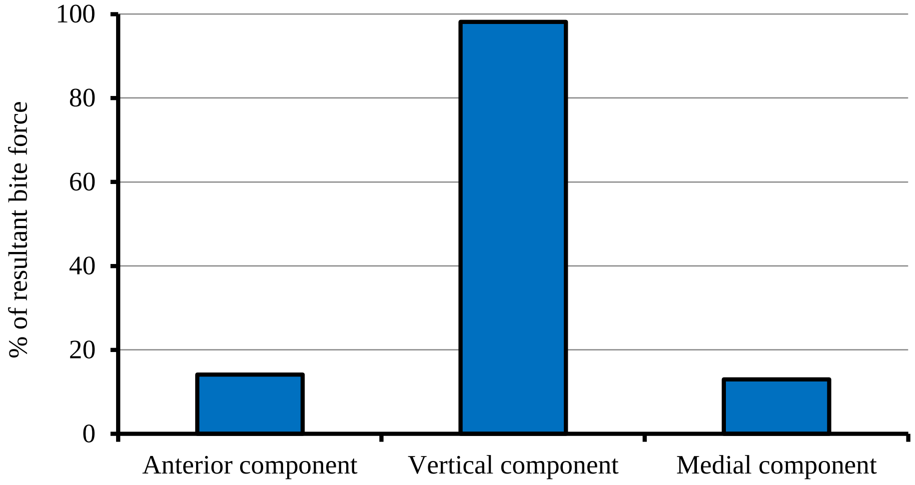

The table below displays the peak activation values of the jaw closer muscles (expressed as a percentage of their maximum force) when simulating premolar crushing. In order to sufficiently compress the food bolus, the activations of muscle Group 1 were slightly larger than those associated with molar crushing, although the activations of muscle Group 2 were similar. This highlights the greater effort required to overcome a resistance to compression as the bite point moves anteriorly along the molar tooth row. Consequently, pre-molar crushing recruited 40.5% of the total muscle force available, which is only 4.9% larger than in molar crushing, but 23.5% less than in incisor biting.

Muscle	Balancing side %	Working side %
Superficial masseter	38.9	7.2
Posterior deep masseter	32.9	100
Ant. Zygo.mandibularis	13.7	60.0
Post. Zygo.mandibularis	27.0	100
Superficial temporalis	52.3	100
Deep temporalis	83.1	66.7
Medial pterygoid	61.4	11.8
Lateral pterygoid	48.0	2.3
